# New melanocortin-like peptide of *E. coli* can suppress inflammation via the mammalian melanocortin-1 receptor (MC1R): possible endocrine-like function for microbes of the gut

**DOI:** 10.1038/s41522-017-0039-9

**Published:** 2017-11-13

**Authors:** Xiaoling Qiang, Anthony S. Liotta, Joseph Shiloach, J. C. Gutierrez, Haichao Wang, Mahendar Ochani, Kanta Ochani, Huan Yang, Aviva Rabin, Derek LeRoith, Maxine A. Lesniak, Markus Böhm, Christian Maaser, Klaus Kannengiesser, Mark Donowitz, Shervin Rabizadeh, Christopher J. Czura, Kevin J. Tracey, Mark Westlake, Aida Zarfeshani, Syed F. Mehdi, Ann Danoff, Xueliang Ge, Suparna Sanyal, Gary J. Schwartz, Jesse Roth

**Affiliations:** 1 Laboratory of Diabetes and Diabetes Related Research, US, USA; 2 Feinstein Institute for Medical Research, Northwell Health System, Manhasset, NY USA; 3 Hofstra Northwell School of Medicine, Hempstead, NY USA; 4 National Institutes of Health, Bethesda, Baltimore, MD USA; 5 Department of Emergency Medicine, Manhasset, NY USA; 6 Icahn School of Medicine at Mount Sinai, New York, NY USA; 7 University of Muenster, Münster, Germany; 8 University Teaching Hospital, Luenburg, Germany; 9 Johns Hopkins University School of Medicine, Baltimore, MD USA; 10 Cedars-Sinai Medical Center, Los Angeles, CA USA; 11 Perelman School of Medicine at the University of Pennsylvania, Philadelphia, USA; 12 Department of Cell and Molecular Biology, Uppsala University, Uppsala, Sweden; 13 Albert Einstein College of Medicine, Bronx, NY USA

## Abstract

*E. coli* releases a 33 amino acid peptide melanocortin-like peptide of *E. coli* (MECO-1) that is identical to the C-terminus of the *E. coli* elongation factor-G (EF-G) and has interesting similarities to two prominent mammalian melanocortin hormones, alpha-melanocyte-stimulating hormone (alpha-MSH) and adrenocorticotropin (ACTH). Note that MECO-1 lacks HFRW, the common pharmacophore of the known mammalian melanocortin peptides. MECO-1 and the two hormones were equally effective in severely blunting release of cytokines (HMGB1 and TNF) from macrophage-like cells in response to (i) endotoxin (lipopolysaccharide) or (ii) pro-inflammatory cytokine HMGB-1. The in vitro anti-inflammatoty effects of MECO-1 and of alpha-MSH were abrogated by (i) antibody against melanocortin-1 receptor (MC1R) and by (ii) agouti, an endogenous inverse agonist of MC1R. In vivo MECO-1 was even more potent than alpha-MSH in rescuing mice from death due to (i) lethal doses of LPS endotoxin or (ii) cecal ligation and puncture, models of sterile and infectious sepsis, respectively.

## Gut bacteria: Helping out with hormones?

A molecule released by the common bacterium *E. coli* may act like a hormone on cells in the gut, with beneficial anti-inflammatory effects. The molecule is a short protein fragment known as a peptide. Its hormone-like activity on cultured mammalian cells was discovered by an international team of researchers led by Jesse Roth at the Feinstein Institute for Medical Research in Manhasset, USA. The peptide shares structural similarities with two known mammalian hormones and binds to an identified hormone receptor molecule. In some circumstances, it was shown to save mice from death by suppressing damaging inflammation. This discovery could broaden our understanding of the beneficial effects of gut bacteria. If applicable in humans it could reveal another link in our subtle relationship with bacteria, and may lead to uses in preventive and therapeutic medicine.

## Introduction

Every mammalian cell is a secretory cell, programed to release a limited menu of hormone-like molecules. Every mammalian cell displays a modest menu of cell surface receptors that recognize and respond to the hormone-like molecules in the extracellular medium. This whole body endocrine-like system provides exquisite coordination for the hundreds of trillions of cells that constitute the host. In an entirely separate communication venue, under the rubric of quorum sensing, bacteria have been shown to have intercellular communication systems by which the microbes release and respond to intercellular communication molecules, providing microbe to microbe exchange of information.

An avalanche of studies in the 21st century have described many, often dramatic examples where mammals and the microbes that reside in their intestines influence each other greatly.^1–4^ Except in a few examples where complex dietary components or small molecules of metabolism, e.g., acetate and butyrate have been implicated, the molecules that mediate this communication have hardly been characterized.^5,6^ In the present paper we introduce, a melanocortin-like peptide spontaneously released from *Eschericia coli* during growth in a simple medium (MECO-1 = melanocortin-like peptide of *E. coli*) that has structure and function in mammalian systems that is close to alpha-melanonocyte-stimulating hormone (alpha-MSH) and adrenocorticotropin (ACTH), the best studied melanocortin hormones of humans, mice, and other mammals.^7–12^ In vitro, in model systems, all three peptides at femtomolar concentrations, acting via the mammalian melanocortin-1 receptor (MC1R), potently inhibit cytokine release in response to pro-inflammatory stimuli. More dramatically, MECO-1 is at least as powerful as alpha-MSH in rescuing mice from death due to sterile (lipopolysaccharide (LPS)-induced) sepsis and polymicrobial (cecal ligation-induced) sepsis. These studies lead us to raise the possibility that microbes resident in the gut produce messenger molecules that can act on mammalian hosts via the hosts’ endogenous hormone signaling pathways to counter the pro-inflammatory stimuli of microbial components and to act much more broadly as a newly recognized endocrine-like organ.

## Results

### Purification of MECO-1

*E. coli* (ATTC 25290), when grown in an elemental medium, rapidly accumulated ACTH immunoactivity in the cells and in the medium (Fig. S-1A). The material from the cells and from the medium were recognized by each of four anti-ACTH antisera (Fig. S-1B). Antiserum 1 and 2, which recognize epitopes at the N-termini and C-termini of ACTH, reacted most strongly with the *E. coli* material. Antiserum 3, which best recognizes the C-terminus of ACTH, and antiserum 4 which best recognizes the mid-portion of ACTH, were 7– 20 fold less reactive.

Cell-free conditioned fermentation medium (300 l), taken through a standard multi-step purification procedure for ACTH, with ACTH immunoactivity as the guide (Fig. S-1C), yielded a single peak of ACTH immunoactivity (Fig. S-1D) whose structure is identical to the 33 amino acids at the C-terminus of elongation factor G (EF-G) of *E. coli*.^10^ Note especially the amino acid sequence overlaps of MECO-1 with the amino-terminal region of alpha-MSH (and ACTH). Also, note the overlaps of MECO-1 and C-terminal regions of ACTH. That MECO-1 tracked so faithfully the path of ACTH through multiple subtle chromatographic steps suggests to us that the surface of the two molecules are much more similar than we might infer from just the amino acid sequence data (Fig. S-1A).

### MECO-1 and alpha-MSH inhibit cytokine release

Macrophage-like RAW 264.7 cells highly express melanocortin-1 receptors (MC1Rs), linked to anti-inflammatory processes.^13^ When incubated overnight, these cells release modest amounts of HMGB-1, a powerful pro-inflammatory cytokine (Fig. S-2). In the presence of LPS (100 ng/ml), HMGB-1 accumulation was tripled. The LPS-induced cytokine secretion was markedly reduced in the presence of MECO-1 or alpha-MSH (Fig. S-2). ACTH 1-39 at the same molar concentrations gave results indistinguishable from MECO-1 and alpha-MSH (data not shown).

**Fig. 1:**
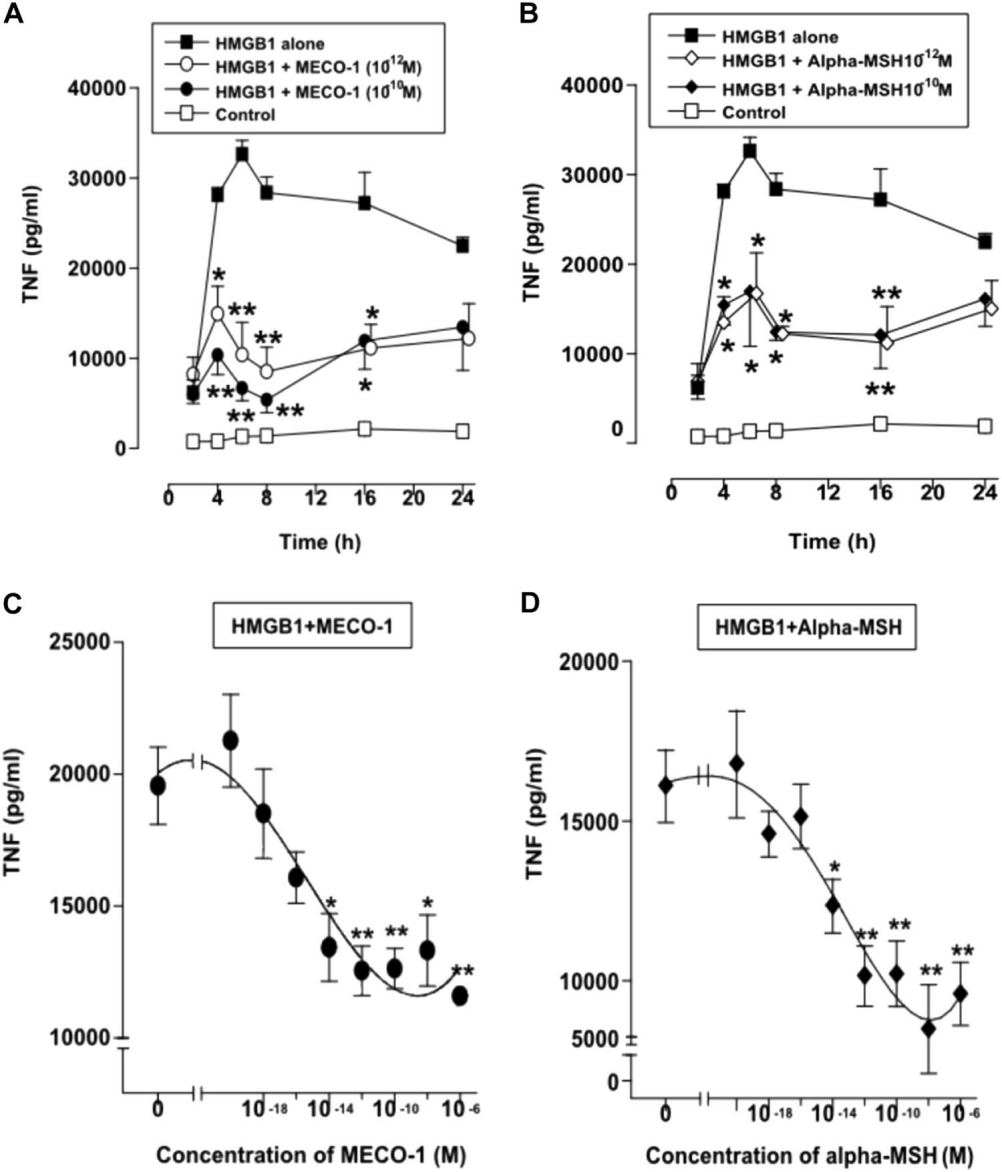
MECO-1 attenuates HMGB1-induced TNF release by macrophages in culture. Murine macrophage-like RAW 264.7 cells were incubated for up to 24 h with purified recombinant HMGB1 (0.1 mg/ml) in the absence or presence of MECO-1 (Panel **a**), or alpha-MSH (Panel **b**), at 10^−10^ and 10^−12^ M. The levels of TNF in the culture medium were determined by ELISA and expressed as mean ± SEM of two independent experiments (in duplicate). *p < 0.05, **p < 0.01 vs. control (“HMGB1 alone”). [In a third experiment (not shown), very similar results were obtained except that the maximum TNF release was observed at 16–24 h, similar to results of others with human endothelial cells]. In panels **c** and **d**, cells were incubated with HMGB1 for 6 h with or without MECO-1 or alpha-MSH (10^−16^–10^−6^ M). Data represent mean ± SEM of three independent experiments performed in duplicate. With 10^−14^ M peptide, results were significant at *p < 0.05, and at 10^−12^ M or more were typically **p < 0.01. In our experiments, at concentrations of MECO-1, alpha-MSH, and ACTH of around 1 nM and higher, dose response curves often reverse direction, i.e., turn upward. The reversal is more clearly seen in Fig. 2. The mechanism of the reversal is not known. (Desensitization, receptor inactivation, receptor internalization, and dissociation of dimeric receptors are all plausible candidates.) As a result, the shape of the lower part of the curve and the actual inflection point may vary from experiment to experiment. This feature may be missed when results of several experiments are combined

With macrophage-like RAW 264.7 cells, HMGB-1 stimulated a rapid multifold increase in the release of tumor necrosis factor-alpha (TNF-alpha). Release of this pro-inflammatory cytokine^14^ was blunted substantially by 1 pM and 100 pM MECO-1 (Fig. 1a) and alpha-MSH (Fig. 1b). The blunting effect of the two peptides on TNF release was observed over the period from 4 to 16 h after addition of HMGB-1 (Fig. 1a, b), with the largest difference detected at 6 h. With 6 h incubations, both peptides showed dose dependence with significant effects noted with both at 10^−14^ M (Fig. 1c, d). ACTH gave very similar results, with highly significant suppression, typically in the subpicomolar range (data not shown).

**Fig. 2:**
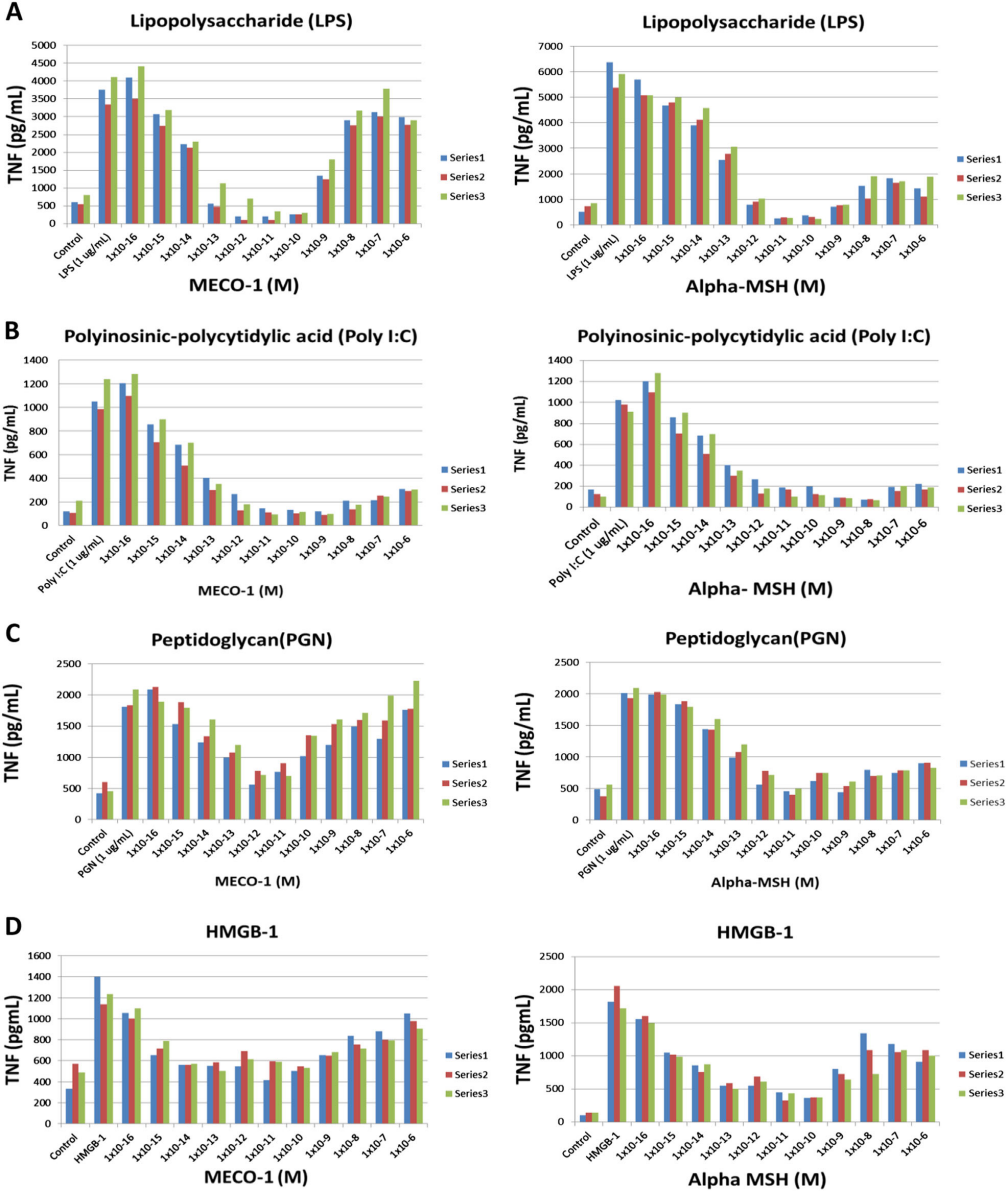
Cytokine-induced TNF release inhibited by MECO-1. **a** Lipopolysaccharide (LPS), derived from the cell wall of Gram-negative bacteria, is a TLR4 and TLR2 agonist. RAW 264.7 cells were treated with MECO-1 and LPS simultaneously and incubated overnight. Supernatants were tested for TNF with ELISA.** b** Poly I:C, synthetic double stranded RNA, is recognized by TLR3. RAW 264.7 cells were pre-treated with MECO-1 and then stimulated with Poly I:C. Supernatants were tested in ELISA by TNF.** c** Peptidoglycan (PGN), another TLR2 agonist, is ubiquitous in the cell wall of bacteria. RAW 264.7 cells were pre-treated with MECO-1 and then stimulated with PGN; after overnight stimulation, supernatants were harvested and tested for TNF ELISA.** d** HMGB-1. Each vertical bar is the mean of three experimental points, using HMGB-1 to induce TNF release. Three sets of three experiments were used for the measurement of inhibition

LPS-induced release of TNF-alpha gave similar patterns of results (Fig. 2a). Attenuation was observed at 10^−15^ M with both MECO-1 and alpha-MSH. A very low valley (∼90% inhibition) was achieved at 10^−11^–10^−10^ M. At peptide concentrations greater than 10^−10^ M the attenuation was lost in a concentration-dependent manner. Poly I:C, which is pro-inflammatory via toll-like 3 receptors (TLR3),^15^ gave similar results. Concentrations from 10^−15^ to 10^−9^ M yielded up to 90% inhibition. MECO-1 and alpha-MSH at concentrations above that had somewhat reduced effects (Fig. 2b). A similar pattern of TNF release was observed with peptidoglycan (PGN), an activator of toll-like 2 (TLR2) receptors.^16^ Up to 75% inhibition was noted in the picomolar range of MECO-1 and alpha-MSH, with waning inhibition at nanomolar concentrations (Fig. 2c). With HMGB-1, an activator of TLR4 (and also TLR2), similar results were obtained with up to 80% inhibition (Fig. 2d).

Overall, the cytokine release by RAW 264.7 cells in response to a wide range of pro-inflammatory agents is reduced by alpha-MSH and MECO-1 at femtomolar and picomolar concentrations. With further increases in concentrations of alpha-MSH or MECO-1, a valley is reached and a partial rebound in cytokine release occurs as peptide concentrations are increased further. The biological basis of the valley followed by the rebound are not known. The melanocortin receptor is a G-protein coupled receptor; receptors in this family can exist as non-covalent dimers and show cooperative interactions. Another possibility is that peptides at these concentrations may dimerize to form ligands with a reduced affinity for receptor-binding sites.

With freshly obtained human peripheral blood mononuclear cells (PBMC), MECO-1, and alpha-MSH caused very similar attenuation of HMGB-1-induced TNF release, with significant effects in the picomolar range (Fig. S-3). ACTH gave very similar results (data not shown). The rebound of effect at high concentrations of peptides was not examined. Note that these preparations of human cells are a mixture of cell types but the results lead us to hypothesize that MECO-1 and alpha-MSH with cells from humans will resemble the MECO-1 and alpha-MSH interactions with MC1R-linked events observed in mice.

### Anti-receptor antibody

**Fig. 3:**
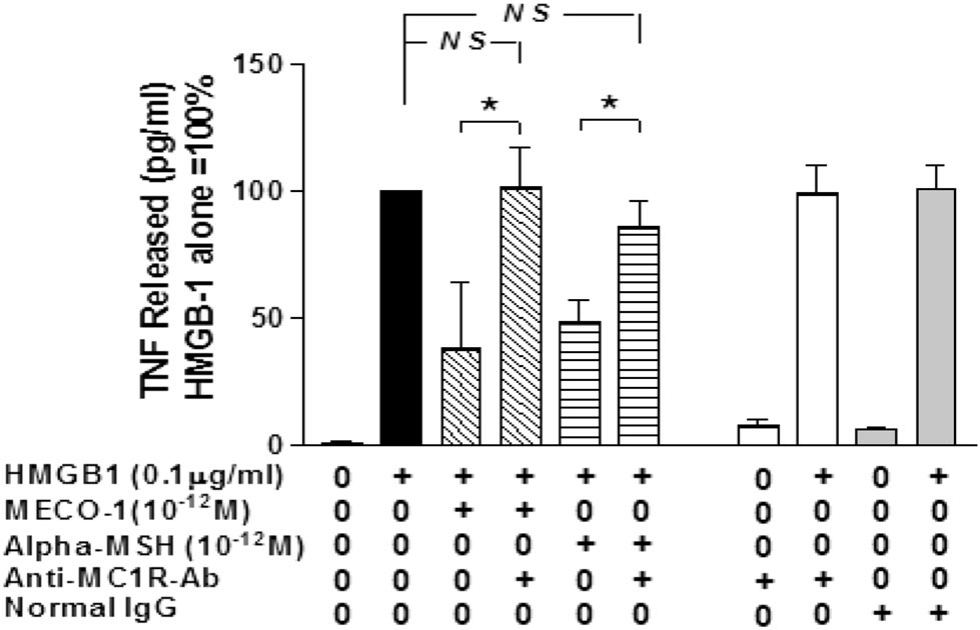
Antibody to MC1R blocks suppressant effect of MECO-1 and alpha-MSH. Murine macrophage-like RAW 264.7 cells were pretreated with specific antibodies (“anti-MC1R Ab”) against melanocortin-1 receptor (MC1R) for 10 min prior to addition of HMGB1 in the absence or presence of MECO-1 or alpha-MSH at 10^−12^ M. At 6 h, the TNF content of the cell-free medium was determined by ELISA, and expressed as mean ± SEM of two independent experiments performed in duplicate. ACTH at the same concentration gave results that were indistinguishable from those of alpha-MSH and MECO-1 (data not shown)

With macrophage-like RAW cells in vitro, an antibody directed against the MC1R (anti-MC1R-Ab) effectively blocked the anti-inflammatory effects of MECO-1 and of alpha-MSH (Fig. 3). MC1R is the receptor implicated as the major mediator of most or all of alpha-MSH effects on macrophage/mononuclear cells, as well as other direct (i.e., extra adrenal) “anti-inflammatory” processes.^17^ ACTH at the same concentration gave results that were indistinguishable from those of alpha-MSH and of MECO-1 (data not shown). Note that with MECO-1 and alpha-MSH, the anti-MC1R anti-serum restored TNF release to equal the levels achieved with HMGB1 alone (in the absence of added melanocortin-related peptides). By contrast, Catania et al. found that their receptor antibody caused TNF release to exceed that induced by LPS alone; they proposed that their macrophages released endogenous alpha-MSH.^18^ Also note that we did not exclude some possible effects of normal IgG on the dampening of HMGB1’s effects by alpha-MSH or MECO-1.

Agouti, the in vivo natural endogenous antagonist of alpha-MSH actions via MC1R, reversed the attenuation by MECO-1 and by alpha-MSH of LPS-induced TNF release. Note that agouti plus LPS raised TNF levels significantly above those observed with LPS alone (Table 1). This is consistent with recent observations that the MC1R receptor alone has a definite basal activity that can be turned off by agouti, providing further evidence that agouti can act as an inverse agonist, with suppression of the basal anti-inflammatory activity of MC1R.^19,20^

**Table 1:** Agouti (endogenous MC1R inverse agonist) inhibits the effects of MECO-1 and alpha-MSH on endotoxin-stimulated TNF release

Agouti (pM)	TNF-accumulated (% of control)	
	MECO-1 (10^-12^ M)	Alpha-MSH (10^-12^ M)
0	52.9 ± 11.2	67.05 ± 10.2
10	134.3 ± 14.5**	132.6 ± 4.9*
10^3^	172.9 ± 10.1**	138.6 ± 12.4*
10^5^	152.4 ± 6.1**	122.2 ± 4.3*

The experiment was as described in the legend of Fig. 3 except that HMGB1 were replaced by LPS (10 ng/ml) and anti-receptor antibody was replaced by agouti, the endogenous peptide that is an inverse agonist of alpha-MSH action via MC1R. TNF release by LPS (10 ng/ml) alone was set at 100%

**p<0.01 vs. MECO-1, *p<0.05 vs. alpha-MSH

### Post-receptor events

The melanocortin receptors, including MC1R, are typically linked via G-proteins to adenylate cyclase, cAMP, and protein kinase A.^19^ In RAW 264.7 cells, ACTH and alpha-MSH stimulated cAMP production in a dose-dependent fashion (Fig. S-4). MECO-1 gave very similar results (Fig. S-4). At the highest concentrations, the three peptides stimulated cAMP almost to the level achieved by forskolin, the alkaloid activator of adenylate cyclase (data not shown). In one experiment, the addition of anti-MC1R antibody shifted the curve three log units to the right for MECO-1-mediated stimulation of cAMP (data not shown). Co-incubation with LPS at 4 ng/ml or with HMGB1 at 100 ng/ml (six experiments) had little effect on the cAMP stimulation (data not shown).

### Protein kinase A

RAW 264.7 macrophage-like cells, when incubated with HMGB1, showed enhanced TNF accumulation. In these experiments the accumulation of TNF was substantially reduced by co-incubation with MECO-1, alpha-MSH, or ACTH at concentrations in the range of 10^−12^–10^−8^ M. The addition of H89, the traditional inhibitor of protein kinase A, abrogated the effects of the three peptides (Fig. S-5). Results were quite similar when experiments were repeated with freshly obtained mononuclear cells from humans (data not shown). A unique interpretation of both sets of experiments is on hold because H89 is now known to also inhibit other enzymes including many kinases.^21^

### NF-kappaB

Activation of macrophages with enhanced cytokine release is one of the many immune system scenarios associated with increases in the activity of NF-kB, a nuclear transcription factor that is a major mediator of many cytokine-related events. MECO-1 and alpha-MSH, under conditions where they attenuated cytokine release from macrophages, were both active in attenuating the rise in NF-kB activity (Fig. S-6). This is in accord with results of others who showed that alpha-MSH-mediated anti-inflammatory effects, mediated via the MC1R, were associated with attenuation of NF-kB activation.

### Microbial analogues of MECO-1

**Fig. 4:**
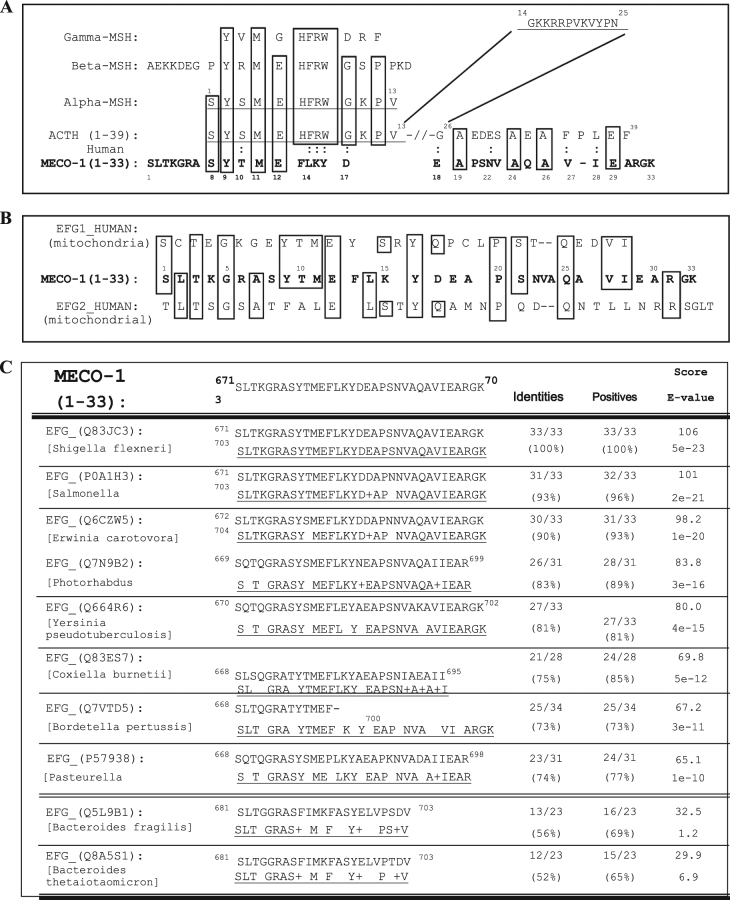
**a** Structure of melanocortins and similar peptides. The four common melanocortins of mammals are derived from one pro-opiomelanocortin (POMC) precursor. ACTH and alpha-MSH are products of alternative processing of the same precursor and have very similar interactions with four of the melanocortin receptors. (The exception is MC2R; in the adrenal MC2R, is the dominant receptor and enables ACTH to have an effect that is orders of magnitude greater than that of alpha-MSH.) While the primary sequences of amino acids 1–13 are identical in ACTH and alpha-MSH, the latter has two post-translational moieties, an N-terminal acetyl and a C-terminal amide, that are important for full potency of alpha-MSH. That MECO-1 lacks the HFRW canonical sequence of the mammalian melanocortins but has bioactivity at MC1R equal to alpha-MSH was a surprise to us. Do note that three of the four substitutions are considered “conservative”. **b** Mitochondrial peptides. The C-termini of the two elongation factors of human mitochondria are compared to MECO-1. Note that each of them is about as close to MECO-1 as each is to the other. **c** Microbial peptides. The first eight sequences are those with the closest matches with MECO-1. The last two are from Bacteriodetes, organisms that are typically highly represented in the human colon

**Fig. 5:**
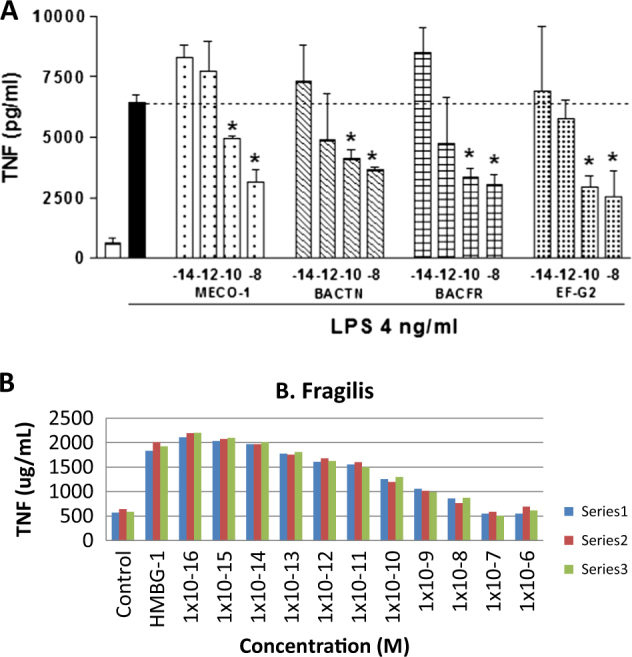
**a** Bioactive analogues of MECO-1. Murine macrophage-like RAW 264.7 cells were incubated with lipopolysaccharide (LPS, 4 ng/ml) in the absence or presence of MECO-1 or BACTN or BACFR, synthetic peptides based on the C-termini of EF-G of *Bacteroides thetaiotamicron* and *Bacteroides fragilis*. EFG-2 refers to human mitochondrial EFG-2 (mtEFG2). For *Bacteroides thetaiotamicron*, we used a 38-mer consisting of residues 681–703 shown in Fig. 4c followed by 704–718 QDKLIKDFEAKQTEE. For *Bacteroides fragilis*, we used a 33-mer consisting of residues, 681–703 shown in Fig. 4c followed by 704–713 = QDKLIKDFES. At 6 h after stimulation, aliquots of cell-free medium were examined. In other experiments, the synthetic peptide based on human mitochondrial EF-G1 (mtEFG1) gave results indistinguishable from EF-G2 (data not shown). The precise structure of the synthetic peptides is indicated in the text (see section “Results” and Table 1). Shown is a representative experiment. Similar results were observed in four other experiments. In this particular experiment MECO-1 showed effects at 10^−10^ M but not at 10^−12^ M. In most experiments, it was effective at 10^−12^ M. *p < 0.05 vs. LPS alone. **b** *Bacteroides fragilis* attenuates TNF release HMGB-1 stimulates release of TNF from RAW cells. Peptide based on structure of C-terminal region of EF-G of *Bacteroides fragilis* inhibited HMGB-1 accumulation so that at 10^−7^ M, TNF release was totally suppressed

MECO-1, the 33 amino acid C-terminus of EF-G of *E. coli*, shares structural similarities with the C-termini of elongation factor-Gs (EF-Gs) of numerous microorganisms, both prokaryotes and eukaryotes (Fig. 4c). As well as with the two EFGs of human mitochondria.^22^ *E. coli* is a minor constituent of the gut microbiota of humans. We tested synthetic replicates of the C-terminus of two more prominent representatives of the microbiota, *Bacteroides thetaiotamicron* and *Bacteroides fragilis* (abbreviated BACTN and BACFR in Fig. 5a); both were about as active as MECO-1 in suppressing TNF release despite many amino acid differences. The *B. fragilis*-based peptide suppressed cytokine release in a manner very similar to results with MECO-1 but without the rebound, i.e., the secondary loss of suppression observed with MECO-1 at high concentrations (Fig. 5b)

### Rescuing mice from sepsis

**Fig. 6:**
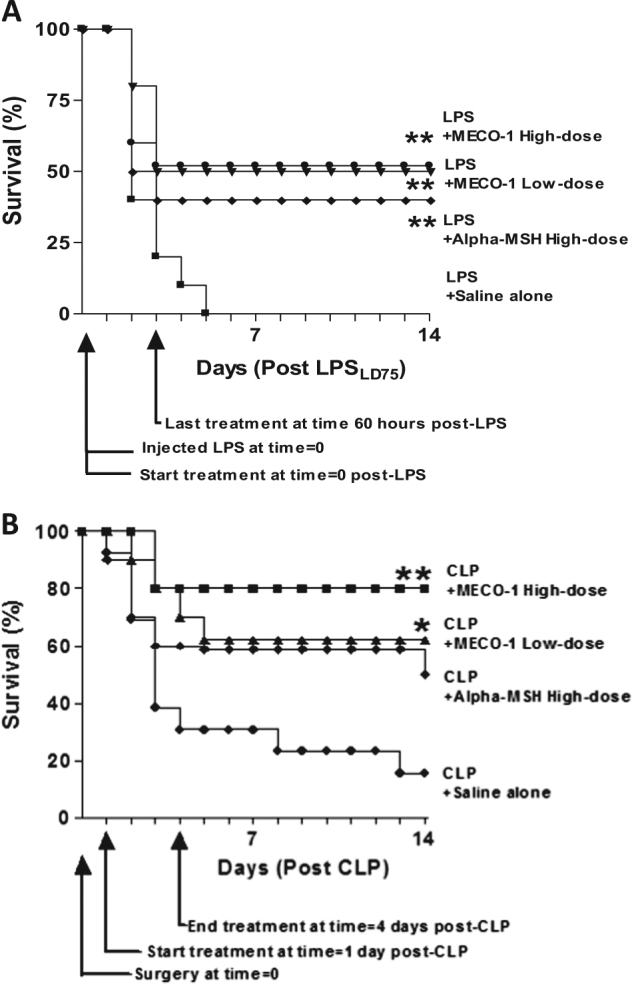
MECO-1 rescues mice from lethal sepsis. **a** Endotoxin. Balb/C mice (males) were injected once intraperitoneally with LPS at a dose estimated to be an LD75. Immediately thereafter, the first doses of MECO-1 at 0.5 mg/kg (low dose), MECO-1 at 5 mg/kg (high dose), alpha-MSH at 5 mg/kg (high dose) or saline alone were administered intraperitoneally. Peptide or saline were given twice daily for 3 days for a total of six doses. Ten mice were in each group. Survival was monitored daily for 2 weeks. The survival benefits of MECO-1 and alpha-MSH were each statistically significant (**p < 0.01-log-rank test) vs. saline treated mice. **b** Cecal ligation and puncture. Balb/C mice were subjected to cecal ligation and puncture (CLP). Starting 24 h post-surgery, treatment was inaugurated, two doses daily for 3 days, for a total of six doses; MECO-1 at 0.5 mg/kg (low dose, *n* = 10) and MECO-1 at 5 mg/kg (high dose, *n* = 10) were compared to saline alone (*n* = 13) and alpha-MSH at 5 mg/kg (high dose, *n* = 10). Animals were monitored for survival for 14 days. The survival benefit of MECO-1 was statistically significant (high dose **p < 0.01 and low dose *p < 0.05 –log-rank test). In this study, one alpha-MSH animal died on the last day of the experiment, so that the benefit of alpha-MSH escaped significance (*p* = 0.08 changed from *p* < 0.05). Note that the high dose of MECO-1 is equal in mg/kg to the high dose alpha-MSH but is less than half on a molar basis. Low dose MECO-1 is ten-fold less than the high dose of MECO-1 and less than one 20th on a molar basis of the high dose of alpha-MSH. On face, our data with CLP (and with LPS) suggest the possibility that in protecting mice from death, MECO-1 may be (up to 30 times) more potent than alpha-MSH, whereas in vitro (see later) they appear to be equipotent. We tentatively propose that the basis for these differences are in the pharmacokinetics

Mice injected with a lethal dose of LPS endotoxin were rescued by the simultaneous administration for 3 days of alpha-MSH or MECO-1, the melanocortin released from *E. coli* (Fig. 6a). In two experiments, none of the saline-treated mice survived [0 of 19], while alpha-MSH rescued 25% [5 of 20], and MECO-1 rescued 50% [15 of 30].

With cecal ligation and puncture (CLP), a mouse model of polymicrobial sepsis similar to perforated appendix with peritonitis, less than 40% of the mice were alive by day 3 and 15% by day 14 (Fig. 6b). Alpha-MSH, as expected, markedly improved survival to around 50% at day 14 (Fig. 6b). MECO-1 was at least as effective (Fig. 6b), so that at a comparable “high” dose, 80% of the mice survived (*p* < 0.01). Injections of the peptides were started at 24 h after surgery and continued through day 4 (administered twice daily for a total of six doses). Overall, in three experiments, 23% of the animals (9 of 39) with CLP polymicrobial sepsis survived. With administration of alpha-MSH, 68% (15 of 22) survived, while with MECO-1, 66% (42 of 64) survived.

**Fig. 7:**
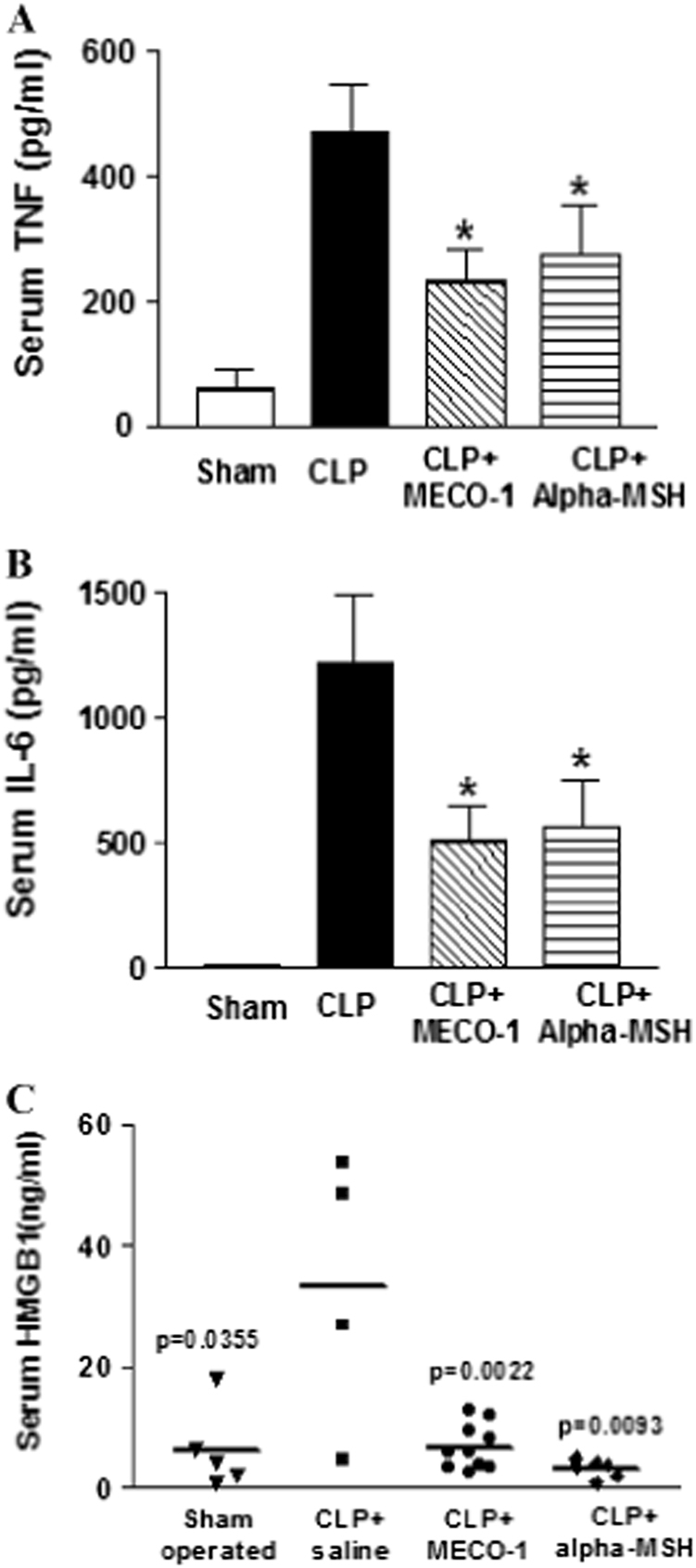
MECO-1 attenuates accumulation in blood of HMGB1 and other cytokines following cecal ligation Balb/C mice were operated (cecal ligation and puncture = CLP) as in Fig. 6b. At 24 h post-surgery they received one dose of saline (*n* = 9) or MECO-1 at 5 mg/kg (high dose, *n* = 10) or alpha-MSH at 5 mg/kg (*n* = 10) intraperitonealy. Survivors at 40 h post-surgery were killed, and blood was obtained for measurement of cytokines (designated protocol Colitis B in section “Methods”). Serum levels of **a** TNF and **b** IL-6 were determined by ELISA, and expressed as mean ± SEM of two independent experiments in duplicate (*p < 0.05, vs. CLP alone). **c** Serum HMGB1 levels were determined by Western blot analysis with reference to standard curves with purified HMGB1. Plotted are levels of HMGB1 in serum for each individual animal. Note that five (of the nine) animals treated with saline alone died before 40 h and their results are not included. We speculate that some of the mice that perished before 40 h may have had cytokine levels that were as high or higher than those observed in those that survived and that differences reported here between CLP vs. CLP + melanocortin peptides are less than might have been observed at an earlier time point

In a parallel experiment (designated protocol B in section “Methods”), sham surgery or CLP was performed. One treatment (peptide or saline) was given at 24 h. At 40 h, serum cytokines were measured (Fig. 7). Only four out of nine of the CLP-operated saline-treated animals survived to 40 h. The survivors had very high serum levels of TNF and IL-6, two cytokines characteristic of the early stages of sepsis (solid bars in Fig. 7). Six of ten alpha-MSH-treated animals and all ten of the MECO-1-treated animals survived to 40 h. In the survivors at 40 h (striped bars in Fig. 7), the animals treated with alpha-MSH and MECO-1 showed significantly muted responses of TNF and of IL-6 (*p* < 0.05). HMGB1, a major cytokine linked to late lethality with sepsis (i.e., at 48–72 h), was elevated in three of four saline-treated CLP animals but in none of those treated with alpha-MSH or MECO-1 (Fig. 7). Because the operated but untreated mice die so swiftly, a detailed time course of cytokine dynamics, although desirable, would have required a very large number of animals.

### Colitis in mice—pilot studies

A widely used model of colitis (induced by dextran sodium sulfate in the drinking water) is ameliorated by parenterally administered alpha-MSH.^23^ That the MC1R melanocortin receptor is playing a significant role in controlling inflammation in the intestine in vivo is supported by the observations that mice null in MC1R (due to a frame shift mutation) are devastated by dextran-induced colitis.^24^ In the pilot study here with dextran-induced colitis (designated colitis A), MECO-1 intraperitoneally markedly attenuated the severe weight loss, a systemic symptom that is typical of colitis in these animals (Fig. S-7), but histological improvement was not noted. In another pilot study (colitis B) with a slightly different protocol, intrarectally administered anti-MECO-1 antibody (but not control serum) aggravated the dextran-induced colitis (Fig. S-8A and S-8B). In aggregate, these data are consistent with but do not prove the hypothesis that in the lumen of the gut, melanocortin ligands, acting on MC1R melanocortin receptors, may be playing a physiological role in limiting inflammatory reactions in the bowel. The mechanisms are unknown by which the colon can maintain the uninflamed state in the presence of such dense collections of microbes that elsewhere would provoke high levels of inflammation. Whether MECO-1 and the MECO-1 equivalents from other intestinal microbes are contributors seems possible but clearly not yet proven. Interestingly, MC1R, which can be activated by alpha-MSH, ACTH, and MECO-I, has been shown to have a functional role in limiting intestinal inflammation; deletion of that receptor produces colitis.^24^

### EF-Gs of mitochondria

The two EF-Gs of human mitochondria, mtEFG1 and mtEFG2, have MECO-1 like sequences (Fig. 4b). Both of these EF-Gs are coded in nuclear DNA but are transported to mitochondria, where they function as elongation factors for mitochondrial production of proteins. The C-termini of the mitochondrial EF-Gs are as similar to each other as each is to the *E. coli* structure (Fig. 1b). Synthetic replicates were made of the C-termini of the EF-Gs of human mitochondria with 28 amino acids for mtEFG1 and 33 amino acids for mtEFG2 as shown in Fig. 4.^22^ Despite many amino acid substitutions, synthetic replicates are each as active as MECO-1 in suppressing TNF release. Data with human mtEFG2 are shown in Fig. 5a; human mtEFG1 gave similar results (data not shown). Recall that apoptosis in mammals is characterized by (i) massive release of proteins from the mitochondria and (ii) a remarkable absence of inflammation. It is not yet known whether the two mitochondrial EFGs contribute peptides that suppress this inflammation.

## Discussion

We have shown here that *E. coli* freshly grown in culture release a 33 amino acid peptide, MECO-1, that has structural and especially functional similarities to alpha-MSH, a major melanocortin hormone of mammals. In their interaction with MC1R, one of the five melanocortin receptors of mammals, the two peptides, alpha-MSH and MECO-1, appear to utilize the same receptor and post-receptor pathways and produce quite similar effects in suppressing cytokine release in vitro and in vivo. Both peptides dramatically rescue mice from sepsis, both sterile (LPS-induced) and polymicrobial (following CLP). Although not yet demonstrated experimentally, we hypothesize that MECO-1 is also released by *E. coli* in the intestine and acts on MC1Rs of immune-related cells of the colon of the host. We hypothesize further that MECO-1 and likely other secretory products from the very dense concentration of microorganisms in the gut (among the highest bacterial cell densities recorded anywhere) collectively produce anti-inflammatory effects that help to maintain the normal un-inflamed state. We suspect that other elements may contribute to this very potent anti-inflammatory chorus: MECO-1-like molecules from other microbes; alpha-MSH from the host; and possible contributions via MC3R receptors.

### MECO-1 is not alone

MECO-1 is but one of seemingly many melanocortin-related peptides from bacteria. During the purification of MECO-1 from *E. coli* conditioned medium, we detected at least two other *E. coli* peptides that, like MECO-1, were reactive with anti-ACTH antibodies but were not purified further. Synthetic peptides whose structures we copied from the C-termini of both mitochondrial EF-Gs and from EF-G of two Bacteroidetes, despite marked differences in structure, were also highly active in suppressing cytokine release from model cells via MC1R-linked pathways. To compare MECO-1 to alpha-MSH in a system independent of both MC1R and the accompanying anti-inflammatory effects, we used the physiological meal terminating effects of alpha-MSH. In vivo in mice and in humans, meal termination is promoted by alpha-MSH that is released from the arcuate nucleus that binds to MC4R of neighboring neurons. This phenomenon is mimicked well by infusing alpha-MSH via cannullae into the hypothalamus of feeding mice; in a preliminary set of experiments MECO-1 gave concentration and time-dependent blunting of feeding that was very similar to that obtained with alpha-MSH (data not shown).

### Possible wider distribution

Traditionally, commensal microbes in the gut (and their products) were believed to be confined to the lumen of the gut. Reviewers of this paper reminded us of studies that have shown that the barriers for passage into regions beyond the lumen of the gut are not uniformly intact.^25,26^ Especially with obesity, viable commensal bacteria are well represented beyond the GI tract, we consider the possibility that hormone-like peptides from these microbes, may under some circumstances, distribute throughout the body like many canonical hormones.

### Historical perspective

Until now, there has been a separation between the intercellular communication provided by endocrine-like systems of mammals and other vertebrates, which first came to light 150 years ago, and the quorum sensing systems of bacteria that have been elucidated over the last several decades. The present work crosses that divide. Peptides produced by bacteria can act on receptors in mammals like peptide hormones of mammals.

The 21st century has witnessed an explosion of understanding of the microbiota and cataloguing of many changes in mammals brought by their microbiota. These changes have been ascribed to nutritional changes^27–29^ or to metabolic intermediates, such as acetate^5^ or butyrate.^6^ Our demonstration of hormone-like messenger peptides from bacteria interacting with specific hormone-related receptors on cells of the mammalian host provides for much better defined specificity of interactions.

### Limitations of the study

We demonstrated MECO-1 in culture medium and in extracts of *E. coli* grown in culture but have not searched for it in microbes in vivo in mammalian hosts. We demonstrated MECO-1 in *E. coli* but not in other bacteria. With the other bacteria (two species of Bacteroidetes) we synthesized plausible MECO-1-like agents based on the structure of MECO-1 and the structure of the C-terminus of the EF-Gs of the Bacteroidetes compared to that of *E. coli*. In experiments where we rescued animals from death (from sterile or polymicrobial sepsis) we administered MECO-1 by parenteral injection, not by installation into the GI tract.

### Is MECO-1 a Rosetta Stone?

In the present study, we show that *E. coli* in culture “spontaneously” releases a 33 amino acid peptide that closely mimics in vitro and in vivo the bioactivity of alpha-MSH, a bona fide 13 amino acid peptide hormone. This melanocortin hormone of mammals and the melanocortin-like peptide native to the bacterium in the femtomolar range are remarkably similar in their suppression of pro-inflammatory reactions in vitro, operating via the same MC1R and its post-receptor pathway. The ability of both peptides to save mice from death due to sepsis, both sterile and polymicrobial, is quite dramatic.

In the last decade, scores of phenomena have been recognized where the microbiota and the host (human or animal) influence one another. The molecular basis for these influences are hardly mapped. In a few studies, metabolic intermediates (e.g., acetate and butyrate) or complex macromolecular mixtures have been implicated but the vast majority of influences are un-defined at the molecular level. We raise the possibility that MECO-1 is an important model for hormone-like signaling between resident microbes and mammalian hosts.

## Materials and methods

### Materials

Recombinant rat HMGB-1 was prepared as previously described,^30^ and passed over a polymyxin B column to remove any LPS contamination; the details of procedures used to free HMGB1 of LPS contamination have been covered elsewhere.^31^ Final LPS content was determined using the Limulus amebocyte lysate assay (BioWhittaker Inc.) as described previously.^31,32^ LPS (*E. coli*, 0111:B4) and macrophage colony-stimulating factor were purchased from Sigma Chemical Co. Anti-MC1R antibody was purchased from (MC1R Antibody (N-19): sc-6875) Santa Cruz Biotechnology Inc. Mouse agouti (93-132)-NH2 was purchased from Phoenix Pharmaceuticals, Inc. ACTH (1-39) and alpha-MSH were purchased from Bachem Bioscience Inc.

### Peptide synthesis

MECO-1 (33 amino acid peptide equivalent to C-terminus of EF-G of *E. coli*) was synthesized and HPLC purified to 88.9% purity in Utah State University Biotechnology Center. LPS was not detectable in the synthetic peptide preparations as measured by Limulus amebocyte lysate assay, as described above.

### Anti-MECO-1 antibody

Synthetic MECO-1 with Freund’s incomplete adjuvant was injected into rabbits at intervals of one or more months. Antibody of the IgG class was affinity purified from serum by using immobilized immunopure protein-A/G column (Pierce). Antibody titers were determined by a direct ELISA in 96-well format. LPS was not detectable in the purified antibody preparations as measured by Limulus amebocyte lysate assay (BioWhittaker Inc.)

### Amino acid sequence comparison of MECO-1

Using computer-assisted database, we compared matching sequences with MECO-1 by using the BLAST network service (http://ca.expasy.org/tools/blast, ExPASy BLAST2 Interface). The sequences of both peptides were aligned, and pairwise percentage similarities were calculated using the William Pearson’s lalign program (using matrix file: BLOSUM 50, gap penalties: −14/−4) (www.ch.embnet.org).

### Animal experiments

In vivo studies were performed in accordance with National Institutes of Health guidelines and with the approval of the North Shore-Long Island Jewish Health System’s Institutional Animal Care and Use Committee (IACUC). In mice, polymicrobial sepsis was induced by surgical ligation and perforation of the cecum, a widely used technique known as CLP. Briefly, male BALB/c mice (7–8 weeks old, 20–25 g) were anesthetized with a mixture of ketamine (100 mg/kg) and xylazine (8 mg/kg) intramuscularly, and a 15 mm midline incision was made to expose the cecum. After ligation of the cecum with a 6.0 silk suture below the junction of the ileo–cecal valve, the ligated cecal stump was perforated once with a 22-gauge needle. The cecum was then gently squeezed to extrude a small amount of feces through the perforation site. The cecum was restored to its normal intra-abdominal position, and the laparotomy was closed with 6.0 silk sutures. Immediately after CLP surgery, all animals were resuscitated with normal saline solution (subcutaneously 20 ml/kg body weight), and given a single dose of antibiotics (primaxin 0.5 mg/kg). In sham operated animals, the cecum was temporarily ligated, but the bowel was not punctured; these animals also received antibiotic treatment and resuscitation fluid. All animals were then returned to their cages with free access to food and water.^32^ At 24 h after CLP, animals were randomly grouped, and received intraperitoneally MECO-1 (0.5 or 5 mg/kg), alpha-MSH, (5 mg/kg), or control vehicle (isotonic saline, 0.2 ml) at 24 h post CLP. In protocol A, peptides (or vehicle) were administered twice daily on day 2, 3 and 4 post surgery. Survival was monitored twice daily for 2 weeks, at which time survivors were killed (CO_2_ asphyxia). In protocol B, a parallel experiment, peptides or vehicle were administered once at 24 h. Survival was monitored twice daily after surgery. Survivors were killed (CO_2_ asphyxia) at 40 h to measure blood levels of selected cytokines.

### Animal experiments (Colitis A)

All mice were maintained and studied according to protocols approved by the Johns Hopkins University Animal Care and Use Committees in accordance with the Association for the Assessment and Accreditation of Laboratory Animal Care International. Ten 4-week-old C57BL/6 mice that were bred at Johns Hopkins University (breeders obtained from the National Cancer Institute) were used for this experiment. The drinking water was supplemented with 2% dextran sodium sulfate (DSS, low dose) from day 0 through day 5. Streptomycin [5 g/L] and clindamycin [100 mg/L] were in the drinking water for the entire duration of the experiment. *B. fragilis* 9343::pFD340 (pFD340 confers clindamycin resistance) was used as the *B. fragilis* strain. Strain 9343 was grown overnight in brain heart infusion broth, washed two times in 0.1 N sodium bicarbonate and suspended to ~10^10^ organisms/ml. On day 5, all mice were inoculated via oral-gastric tube with 0.2 ml of *B. fragilis* under ether anesthesia. Each mouse was given intraperitoneal injections twice a day of either MECO-1 peptide (5 mg/kg/dose in PBS) or PBS on days 0 through 7 followed by once daily MECO-1 or PBS injections from days 8 through 11. Mouse weights were recorded daily between 7 and 10 am (Fig. S-8A **)**. Mice were observed at time of injection for activity level and signs of clinical illness. Stool was obtained from each mouse on day 11 and serial dilutions cultured on brain heart infusion plates with clindamycin to quantitate strain 9343 colonization. On day 11, all surviving mice were sacrificed (CO_2_ asphyxia). Cecum and colon (latter Swiss rolled) were preserved in 10% formalin prior to histologic examination by hemotoxylin and eosin staining of 5 μm sections (Fig. S-8B **)**.

### Animal experiments (Colitis B)

Studies were approved by the animals use committee of the University of Muenster. The mice for these experiments were C57BL/6 (Charles River, Germany) housed under pathogen-free conditions at 24 °C with a controlled 12 h day–night cycle and free access to standard diet and drinking water. The mice were 6–8 weeks of age at the beginning of the present study. To induce colitis mice were given 2.5% dextran sodium sulphate (DSS, ICN Biomedicals Inc., Germany) in the drinking water for 6 days. Consumption of drinking water was monitored. Starting from day 1 of dextran sulfate, mice were treated daily with 200 µl of anti-MECO-1 antiserum or a control rabbit serum by rectal application using a gavage needle. To assess disease activity, mice were weighed daily. At day 6 of dextran sulfate-treatment mice were sacrificed (by cervical dislocation) and the colons removed. The organs were opened longitudinally; small sections were taken from the proximal and distal part of each colon for analysis of myeloperoxidase activity. The remaining tissue was embedded as “swiss rolls” in O.C.T. compound (Tissue-Tek, Sukura Fine Tek Europe, NL) and kept frozen at −80 °C until further use. Sections of 5 µm were stained with hematoxylin and eosin. Histological analysis focused on epithelial denudation, ulceration, edema, and leukocyte infiltrates (Fig. S-8B).

### Analysis of myeloperoxidase levels

Myeloperoxidase activity was measured in samples of colon as an indicator of leukocyte accumulation. After snap-freezing in liquid nitrogen, tissue samples were homogenized and suspended in 500 µl of 100 mM NaCl, 20 mM Tris pH 7.5 and 0.1% Triton X-100 (Sigma Chemical Co., Germany). The homogenates were centrifuged at 12,000 rpm for 20 min and the supernatant removed for assay of myeloperoxidase activity. Ten microliters of supernatant were added to 200 µl of 50 mM phosphate buffer pH 6.0 containing 0.4 mg/ml of substrate O-phenylenediamide (Sigma Chemical Co., USA) and 10 µl H_2_O_2_. After 20 min 50 µl of 0.4 mM H_2_SO_4_ were added to stop the reaction. Absorbance was measured at 460 nm and enzyme activity calculated using a standard curve (Fig. S-8C).

### Cell cultures

Murine macrophage-like RAW 264.7 cells were obtained from American Type Culture Collection, and were grown in Dulbecco’s Modified Eagle’s Medium (DMEM, Life Technologies) containing 10% (vol/vol) heat-inactivated fetal bovine serum (FBS, Hyclone Lab. Inc.), penicillin 100 U/ml, and streptomycin 100  μg/ml (BioWhittaker Inc.). Cells were suspended in medium and incubated in 24-well or 48-well tissue-culture plates overnight in a humidified incubator (37 °C, 5% CO_2_). Growth medium was removed and replaced by Opti-MEM I serum-free medium (Life Technologies) overnight. In the experiments, cell monolayers were stimulated with HMGB1 or LPS, in the absence or presence of MECO-1 or alpha-MSH; cell-free supernatants were assayed for TNF by ELISA or HMGB1 by western blotting.

### Human PBMC

Human PBMC were isolated by density gradient through Ficoll-Paque^TM^ PLUS centrifugation (Amersham Pharmacia Biotech), and suspended in RPMI 1640 containing 10% (vol/vol) heat-inactivated human serum (BioWhittaker Inc.), penicillin 100 U/ml, and streptomycin 100 μg/ml, and incubated at 37 °C in a humidified incubator with 5% CO_2_ atmosphere overnight. Non-adherent cells were discarded. Adherent monocytes were washed twice with PBS and were then suspended in 12-well and 24- well tissue culture plates and incubated in the same medium but now enriched with 2.5 ng/ml macrophage colony-stimulating factor for 5–7 days.^33^ The growth medium was removed and replaced by Opti-MEM I serum-free medium overnight. Cell monolayers were incubated with HMGB1, in the absence or presence of MECO-1 or alpha-MSH, and supernatants were assayed for TNF.

### Western immunoblotting analysis

The levels of HMGB1 in the culture medium or murine serum were measured by western immunoblotting as previously described.^30^ Western blots were scanned with a silver image scanner (Silverscaner II, Lacie Limited). The relative band intensity was quantified by using the NIH image 1.59 software. The levels of HMGB1 (expressed as % maximum effect) were calculated with reference to standard curves generated with purified rHMGB1 and expressed as mean ± SEM of four experiments.

### Cytokine assay

Concentrations of TNF and IL-6 were each determined using a commercially available enzyme-linked immunosorbent assay (ELISA) kit (R&D System Inc.) as previously described.^34^ The levels of TNF or IL-6 were calculated with reference to standard curves.

### Cyclic-AMP determinations

RAW 264.7 cells were grown to confluence in culture plates, harvested by classical methods, and then seeded onto 48-well plates (1 × 10^6^/ml). Cells were incubated with peptide at 37 °C in Opti-MEM I serum-free medium containing 1 mM 3-isobutyl-1-methylxanthine. At 30 min, cells were lysed in the original wells. The lysates were extracted from the wells, and cyclic-AMP measurements carried out using the Biotrak kit (cyclic-AMP Biotrak Enzymeimmunoassay System, Amersham Biosciences) including a standard cyclic-AMP curve as recommended by the suppliers.^35^

### NF-kappa-B activity measurement

Nuclear extracts were prepared from 1 × 10^6^ treated cells using the NE-PER nuclear extraction reagents (Pierce). DNA was biotin labeled using the biotin 3ʹ-end-labeling kit (Pierce) in a 50-µl reaction buffer and 5 pmol of double-stranded NF-kappa-B oligonucleotide (5ʹ-AGTTGAGGGGACTTTCCCAGGC-3ʹ and 3ʹ-TCAACTCCCCTGAAAGGGTCCG-5ʹ, Promega). Electrophoretic mobility shift assays for NF-κB was performed using the Lightshift Chemiluminescent Electrophoretic Mobility Shift Assay kit (Pierce) following the manufacturer’s protocol. Densitometric values for the active transcription factors were obtained using GS-800 Calibrated Densitometer Software (Biorad).^36^

### Statistical analysis

Kaplan–Meier analysis was used to determine statistical significance of the differences in survival of mice. *p* ≦ 0.05 was considered significant. Values in the figures were expressed as mean(±)S.E.M. of two or three independent experiments, where each experimental point was derived from duplicates or triplicates. Student’s two-tailed *t*-test was used to compare the means between groups. *p*-value of 0.05 or less was considered statistically significant. The design (including sample size) of experiments in Figs. 2–7, and Table 1 followed closely the design of previous experiments by us and others. The results of prior experiments in our laboratory (both in vitro experiments and animal experiments) were used to decide sample size for each of the experiments shown.^37–41^

The experiments in Supplementary Figs. S-2, S-3, S-4, S-5 and S-6 were performed in our lab. The design of these was based on results of previous experiments. The experiments in Figs. S-7 & S-8 were performed in labs of our collaborators, using designs found previously by them to be successful.

In animal studies shown in Figs. 6a, b, 7a–c sample sizes chosen were based on results of similar experiments performed previously in our lab. No formal randomization was performed and no blinding of the experimenters. We excluded any animal whose body weight was far outside of the range of others or appeared ill.

### Data availability

The authors declare that the data supporting the findings of this study are available within the paper and its supplementary published material.

## Electronic supplementary material

Supplemental Figures
