# Supplementary material for: New melanocortin-like peptide of E. coli can suppress inflammation via the mammalian melanocortin-1 receptor (MC1R): possible endocrine-like function for microbes of the gut
Source: NPJ Biofilms Microbiomes. 2017 Nov 13;3:31. doi: 10.1038/s41522-017-0039-9 (PMC5684143; doi:10.1038/s41522-017-0039-9)
Supplement: Supplementary file 1 — Supplemental Figures [file 41522_2017_39_MOESM1_ESM.docx]

**SUPPLEMENTAL FIGURES**

**Figure S-1A**

**
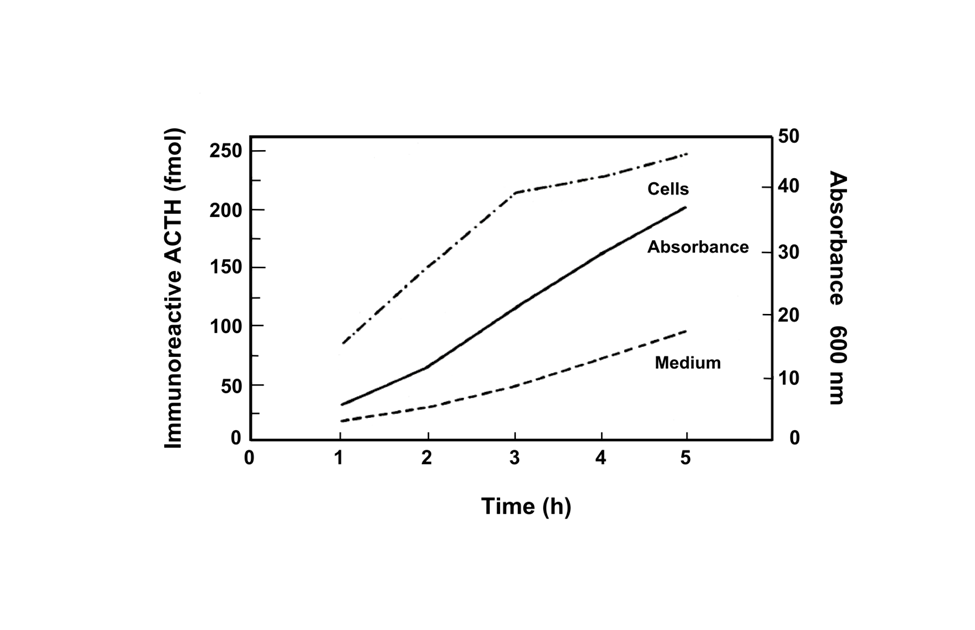
**

**Figure S-1B**

**
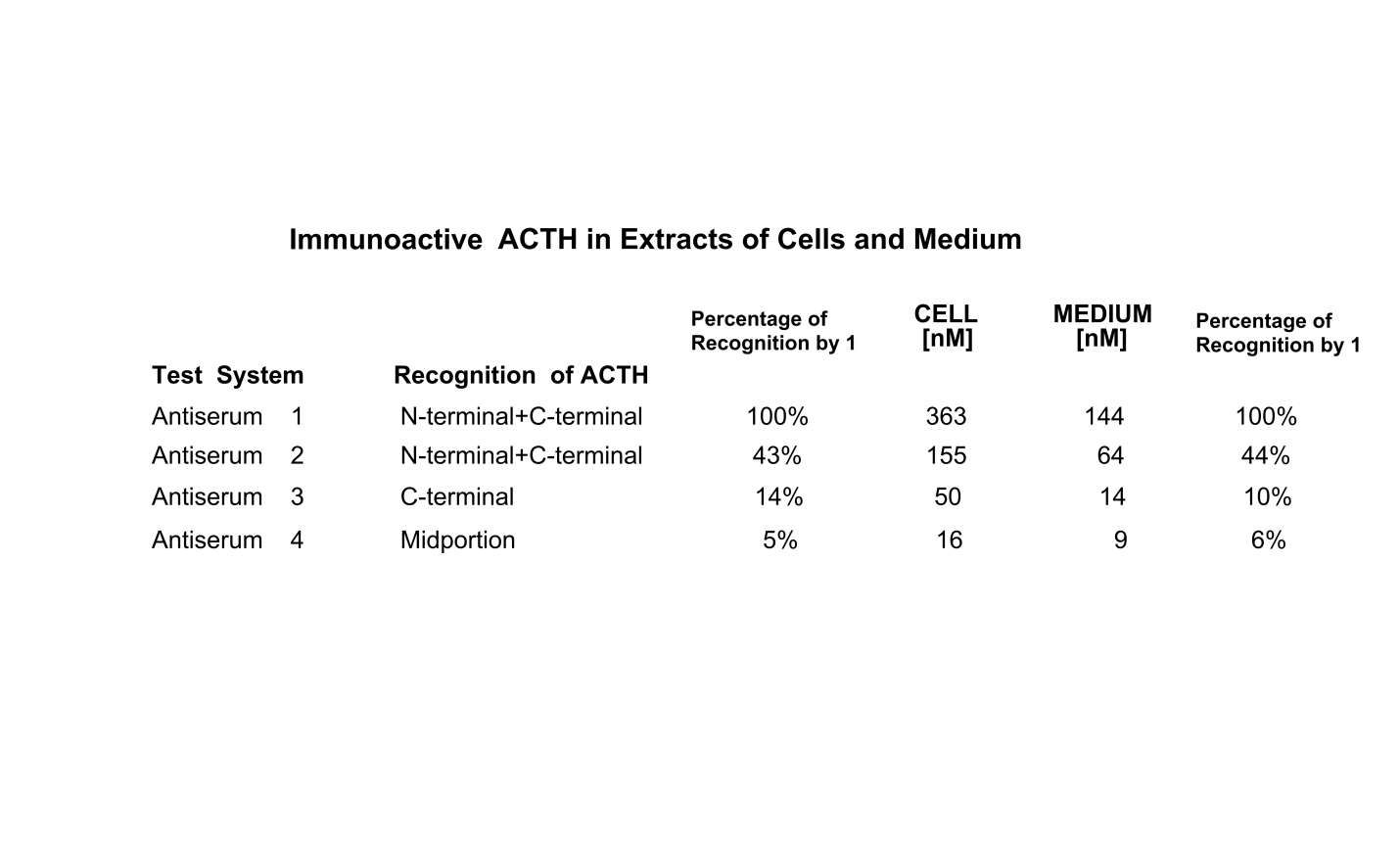
**

**Figure S-1C**

**
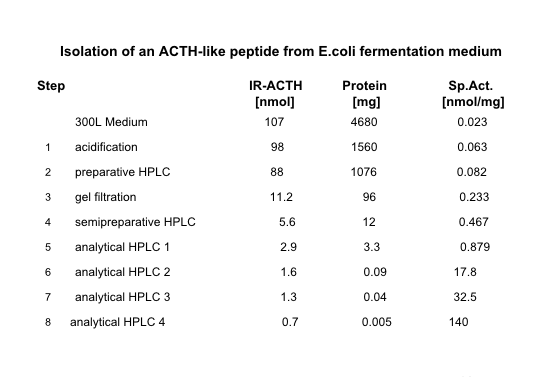
**

**Figure S-1D**

**
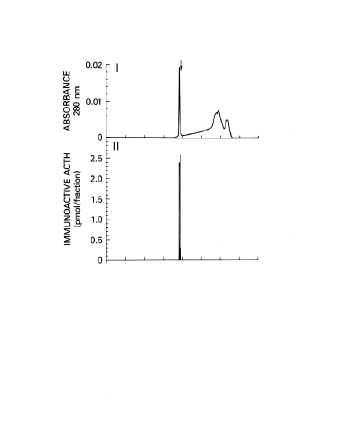
**

**Figure S-2**

**

**

**

**Figure S-3**

**Figure S-4**

**Figure S-5**

**Figure S-6**
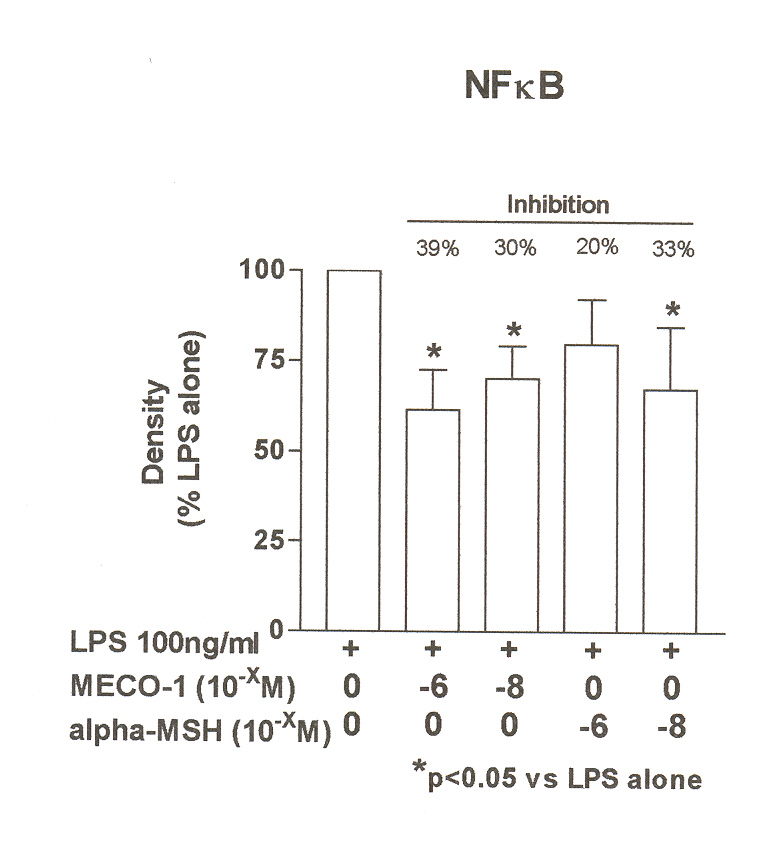


**Figure S-7**

Day of *B. fragilis* inoculum

B. fragilis inoculum

**

*

***

MECO-1 (N=5)

=MECO-1

VEHICLE (N=5)

Weight Change (g)

Days Post Initiation of Dextran

Weight Change of Mice Pretreated with 2% DSS, Inoculated p.o. with B. fragilis and Injected i.p. with MECO-1 or Control Vehicle (Colitis A in text)

**Figure S-8A**


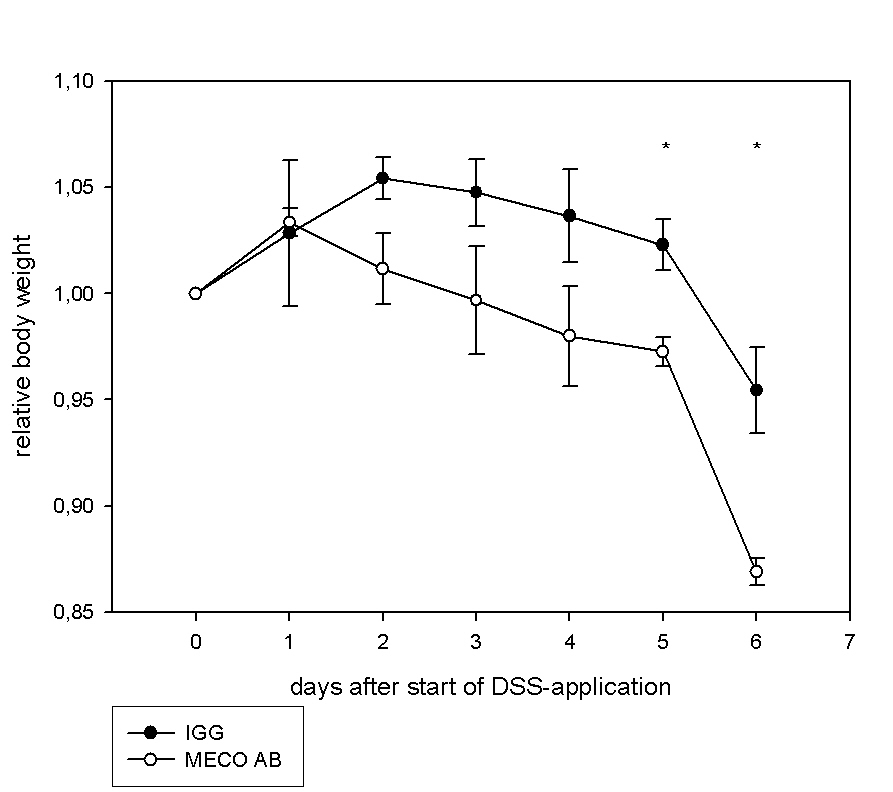


**Figure S-8B**


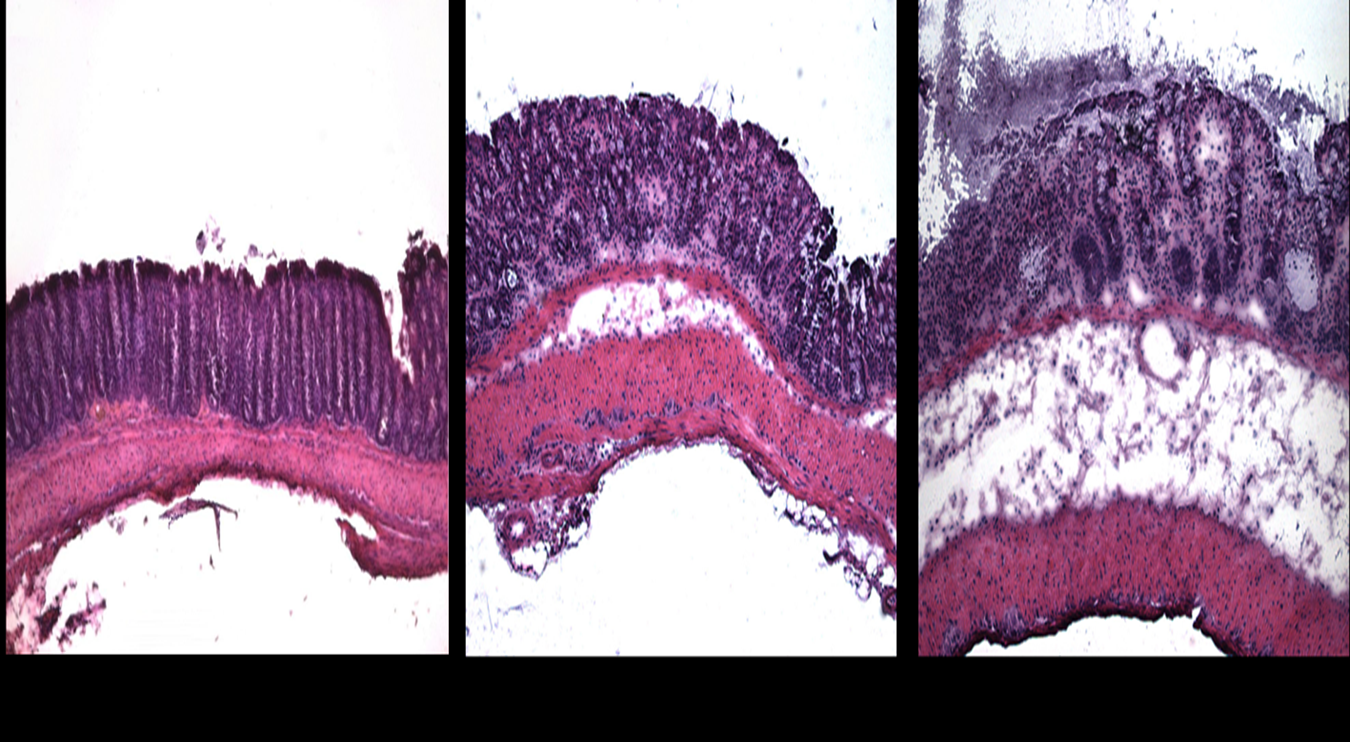


**Figure S-8C**

**
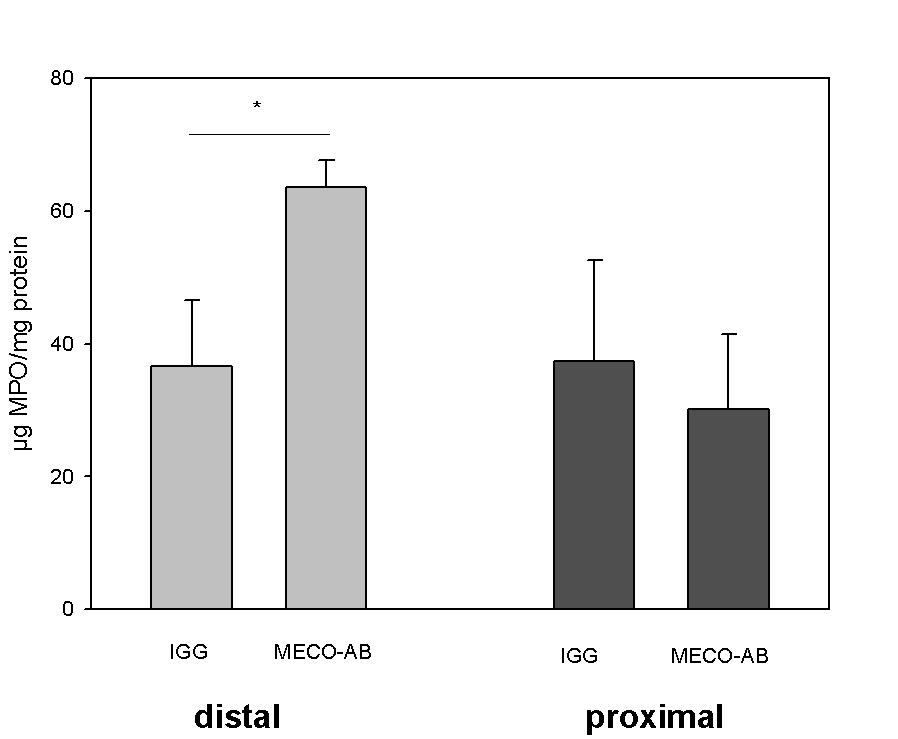
**

**SUPPLEMENTAL LEGENDS**

**Figure S-1. Purification of immunoactive ACTH-related material**

**A, B)** E. coli ATCC 25290 was grown in a simple totally synthetic medium at 37 C pH 6.95 as previously described (37). Using the nonequilibrium immunoassay procedure described previously (38), we detected immunoactive ACTH in conditioned medium and in extracts of cells (Figs S-1A and S-1B). Antiserum 1 & 2, known to be most highly reactive with epitopes at the N- and C- termini of ACTH (1-39) reacted most strongly. An antiserum that reacts strongly with structures at the ACTH C-terminus alone (antiserum 3) or an antiserum that is most reactive with the midportion of ACTH (1-39) were considerably less reactive with our E. coli derived material. Results with cell-free medium were quite similar to results with the cell extracts. Antiserum 1 was employed to monitor immunoactive ACTH-related material at all subsequent stages of the isolation procedure.

**C)** Because it was technically easier to process, the conditioned medium was the starting material to isolate the substance(s) responsible for the ACTH-related immunological activity of E. coli. Medium was adjusted to pH 3.5 with 10N HCl. (Fig S-1C). With storage overnight at

4 C, a precipitate formed that was discarded; it contained little immunoactivity but about 70% of the total protein. The supernatant was filtered through a Durapore tangential filter and pumped onto a PrepPak 500 C­18 reverse phase cartridge using a Waters Associates preparative HPLC system. The column was eluted first with 20% CH_3_CN/13mM trifluoroacetic acid and then with 60% CH3CN/13mM trifluoroacetic acid. The 60% fraction, which con­tained most of the immunoactive ACTH, was processed further. This fraction was concentrated and submitted to gel filtration on a Sephadex G­50 SF (5X100 cm) column, as described previously (39). The fractions containing immunoactive ACTH-related material that eluted with the approximate Kav of human ACTH were pooled, extracted using Sep­Pak C­18 cartridges (39,40), and submitted to seven steps of reverse phase HPLC using gradient elution conditions that we have previously used in other studies (39-41). Briefly, the column and solvent systems used were: step 1, Altex C­18, 10X250mm, 0.4M triethylammonium phosphate/CH3CN; step 2, Altex C­8, 4.6X150mm, triethylammonium phosphate/CH3CN; step 3, Waters C­18 uBondapak, 3.9X300 mm, 0.19% heptafluorobutyric acid/CH3CN; step 4, Waters Phenyl uBondapak, 3.9X300mm, heptafluorobutyric acid/CH3CN; step 5, Waters C­18 uBondapak, 3.9X300mm, heptafluorobutyric acid/CH3CN/0.2M [NH4]2SO4/CH3CN; steps 6 and 7, C­18 uBondapak, 2X300mm, 0.08% trifluoroacetic acid/CH3CN. Four separate batches of medium, ranging from 100 to 600 liters were processed through this procedure. Overall, we estimate a 10,000 fold purification (Fig S-1C). The final product yielded a single peak of immunoactivity. The amino acid sequence of the peptide showed many similarities to alpha-MSH and even more similarities to ACTH. The known specificity of the four anti-sera in Fig S-1B predicted well the observed structure of the microbial peptide (Fig 1A). All of the further characterization studies were performed with synthetic replicates of this peptide, designated MECO-1 (melanocortin from E. coli-1). At very early stages of the purification, we noted two other less abundant ACTH-related peptides that were not studied further.

**Figure S-2. MECO-1 in vitro inhibited LPS-induced HMGB1 release.**

Murine macrophage-like RAW 264.7 cells were incubated with LPS (100 ng/ml) in the absence or presence of MECO-1 or alpha-MSH at 10^-10^ M. At 16-20 hrs after stimulation, aliquots of cell-free medium were examined. HMGB1 levels in the (conditioned) culture medium were expressed as mean ± SEM of four independent experiments with the peak level (obtained with LPS) set at 100%. The reductions in HMGB1 by MECO-1 and by alpha-MSH were significant (** P < 0.01).

**Figure S-3. MECO-1 attenuates HMGB1-induced TNF release by human macrophages in culture.**

Human peripheral blood mononuclear cells (PBMC) were isolated by density gradient centrifugation through Ficoll-PaqueTM PLUS, and cultured for 5-7 days in medium containing macrophage colony-stimulating factor (2.5 ng/ml). The differentiated human macrophages were subsequently incubated for 6 hours with HMGB1 (0.1mg/ml) in the absence or presence of MECO-1 or alpha-MSH at indicated concentrations. The content of TNF in the cell-free medium was determined by ELISA and expressed as mean ± SEM of three independent experiments performed in duplicate. *p< 0.05, **p< 0.01 vs. control (=HMGB1 alone without added melanocortin peptide).

**Figure S-4. MECO-1 stimulation of cyclic-AMP production.**

RAW 264.7 cells were incubated with peptides for 30 minutes at 37 degrees. Cell lysates were extracted and c-AMP measurements carried out using a commercial kit [c-AMP biotrack-immunoassay system, Amersham Biosciences] with a c-AMP standard curve as recommended by the suppliers. In the four experiments with alpha-MSH, stimulation that was statistically significant was noted at 10^-9^ M three times and once at 10^-10^ M. MECO-1 gave positive results at 10^-10^ M in two experiments and at 10^-9^ M and 10^-8^ M in additional experiments. The only experiment with ACTH is shown above. (Effects of LPS at 4 nanograms per ml. or of HMGB1 at 0.1 µg. per ml. on ligand-stimulated c-AMP accumulation were quite modest and inconsistent). The data here are concordant with observations in many other systems where the sensitivity to ligand when measuring a biological end point response is one or more log units to the left of the standard curve for c-AMP production, which has been interpreted to indicate that downstream pathways are exquisitely sensitive to low levels and small changes in c-AMP concentrations.

**Figure S-5. Inhibition of MECO-1 action by inhibition of protein kinase A.**

RAW 264.7 cells were incubated for 6h with HMGB1 (0.1mg/ml) in the presence or absence of melanocortin peptides at 10^-10^ M under conditions described in legend to Figures 2C and 2D. H89 was present at 1mM (added 10 minute before other reagents). Data represent mean +/- SEM of two independent experiments performed in duplicate (**p<0.01).

**Figure S-6. Effect of melanocortin peptides on NF-kappa B activity.**

RAW 264.7 cells (10^-6^) were incubated with LPS (100 ng/ml) with or without MECO-1 or alpha-MSH at 10^-8^ M and 10^-6^ M for 2 hours. Nuclear extracts were mixed with biotin-labeled oligonucleotides containing binding sites for NF-kappa B and then run on a gel, 4-20% Ready gel TBE, in an electrophoretic mobility shift assay. Data represent mean +/- SEM of two separate experiments.

**Figure S-7. MECO-1 ameliorates weight loss with dextran-induced colitis (Experiment =**

**"Colitis A").** Mice were given 2% dextran sodium sulfate (low dose DSS) in their drinking water for five days to induce colitis, during which time all mice were active and gained weight. On day five, they received an enteric inoculum of an enteroxin producing Bacteroides fragilis. The mice also received saline [vehicle] or MECO-1 intraperitoneally daily for all 11 days of the experiment. MECO-1-treated mice exhibited significantly greater weight gain at day 8 (** p<0.03), day 9 (*** p<0.025), and day 10 (* p<0.04) compared to saline treated control mice. The weight changes observed in the saline vehicle group here are typical for mice with this model of colitis. Two of 5 mice in the vehicle-treated group developed diarrhea 48 hours post-inoculum but none in the MECO-1 group. One of 5 mice in the vehicle-treated group died 96 hours post-inoculum. Day 11 stool cultures demonstrated 1010 colonies of Bacteroides fragilis/gram stool weight in both groups. All surviving mice (N=4 control and N=5 MECO-1 treated) were sacrificed on day 11. Severe colitis was evident in 2 of 4 saline control mice and in 3 of 5 of the MECO-1 treated mice. Overall, cecal histology did not differ between the two groups (data not shown). Inflammation score ± SD (0 [normal] to 3 [severe]) was 1.9 ± 1.0 for the MECO-1 mice and 2.1 ± 0.8 for the saline control mice (P=NS). We tentatively concluded that MECO-1 administered systemically (i.p.) ameliorated systemic effects of colitis (i.e. weight loss) but not the enteric process (i.e. histology).

**Figure S-8. Anti-MECO-1 antibody aggravates dextran-induced colitis (Colitis B) –**

C57BL/6 mice were given 2.5% (w/v) dextran sodium sulfate (DSS) in their drinking water throughout the study. Animals of group 1 were, in addition, treated daily starting at day 0 with anti-MECO-1 anti-serum (“MECO AB”) by rectal gavage, while group 2 (colitis controls) received the same volume of control immunoglobulin. After six days, mice were sacrificed and colonic tissue was removed.

**(A) Weight loss associated with colitis was aggravated by anti-MECO AB.** Differences were statistically significant on days 5 and 6.

**(B) Histology**-- At the end of the experiment, the normal pattern of mucosa in the distal colon (left panel) was disrupted as expected with the dextran-induced colitis (center panel) including ulcerations and inflammatory cell infiltrates in the distal colon. Mice treated with anti-MECO-1 antibody (right panel) showed more pronounced and severe ulcerations with areas of epithelial denudation, more intense immune cell infiltration and submucosal edema.

**(C) Myeloperoxidase activity in colonic tissue**. The excised colon was divided into several small parts, which were used for analysis. Significantly more myeloperoxidase (MPO) activity, expressed per mg total protein, was detected in tissue from distal colon of anti-MECO-1 antibody treated mice than in mice treated with control IgG (p<0.05; student’s t-test; n=5 mice/group), reflecting greater density of leukocytes. In samples from the proximal colon, we found no difference between the two groups.
